# An Experimental and Computational Study of Effects of Microtubule Stabilization on T-Cell Polarity

**DOI:** 10.1371/journal.pone.0003861

**Published:** 2008-12-08

**Authors:** Arie Baratt, Sergey N. Arkhipov, Ivan V. Maly

**Affiliations:** Department of Computational Biology, University of Pittsburgh School of Medicine, Pittsburgh, Pennsylvania, United States of America; New York University School of Medicine, United States of America

## Abstract

T-killer cells eliminate infected and cancerous cells with precision by positioning their centrosome near the interface (immunological synapse) with the target cell. The mechanism of centrosome positioning has remained controversial, in particular the role of microtubule dynamics in it. We re-examined the issue in the experimental model of Jurkat cells presented with a T cell receptor-binding artificial substrate, which permits controlled stimulation and reproducible measurements. Neither 1-µM taxol nor 100-nM nocodazole inhibited the centrosome positioning at the “synapse” with the biomimetic substrate. At the same time, in micromolar taxol but not in nanomolar nocodazole the centrosome adopted a distinct peripheral rather than the normally central position within the synapse. This effect was reproduced in a computational energy-minimization model that assumed no microtubule dynamics, but only a taxol-induced increase in the length of the microtubules. Together, the experimental and computational results indicate that microtubule dynamics are not essential for the centrosome positioning, but that the fit of the microtubule array in the deformed body of the conjugated T cell is a major factor. The possibility of modulating the T-cell centrosome position with well-studied drugs and of predicting their effects *in silico* appears attractive for designing anti-cancer and antiviral therapies.

## Introduction

T-killer cells of the immune system form conjugates with cells infected by viruses, as well as with tumor cells, and eliminate them via directed discharge of toxic compounds. The directionality is essential for the effectiveness of killing the intended target as well as for sparing healthy bystander cells, i.e. for specificity of cellular immune response [1]. The killing apparatus is structurally associated with the Golgi apparatus and with the centrosome at the center of convergence of the microtubule fibers of the T-cell cytoskeleton. Polarization (positioning) of this organelle complex in the T cell to the interface with the target cell [2], [3] is recognized as the cell-structural basis of the directionality of cellular immune response [1]. Other types of cell-cell interactions in the immune system similarly involve centrosome polarization [1].

The mechanism of centrosome positioning in T cells has not been established. It appears to be a form of rearrangement of the microtubule cytoskeleton. Other types of microtubule cytoskeleton rearrangements, for example during cell division, proceed to a large degree through disassembly and re-assembly of individual microtubules, which are termed microtubule dynamics. Microtubule dynamics is therefore a foremost candidate for the driving force of the centrosome polarization in T cells, or at least for an essential facilitating mechanism. This view has its most direct support in two experimental studies, which uncovered signal transduction pathways in T cells that might lead to promoting, alternatively, microtubule assembly and disassembly [4], [5]. Earlier studies on primary cytotoxic T-lymphocytes, however, established that the centrosome polarization was insensitive to treatment with taxol, a drug used to suppress microtubule dynamics [6]. Thus, the existing data on the role of microtubule dynamics in T cell polarization appear contradictory.

In the present work, we have examined the sensitivity of polarization to inhibitors of microtubule dynamics in an experimental model that replaces the target cell surface with the optical glass surface coated with a stimulatory clone of antibodies to the T cell receptor. This experimental model has been widely used in cellular immunology because it permits reproducible stimulation of large numbers of T cells and facilitates microscopy data collection and analysis [7]–[16]. Cultured T cells of the Jurkat line that are used in the present study have been previously shown to exhibit the same polarization response to this type of biomimetic surface [9], [12]–[16] as the primary T-killer cells to their immunologically cognate targets.

Experimental observations are further compared in the present work with computational predictions. We have previously been able to explain the polarized location of the centrosome in conjugated T cells as arising from whole-cell structural optimization. The optimality was postulated to be multiobjective, as expressed in the several terms in the empirical energy function that is minimized: The model cell minimizes microtubule bending and cell surface area, while maximizing the area of contact with the target and maintaining the cell volume [13]. This approach is an extension of the energy-minimization method originally used by Holy et al. to explain experiments on microtubule asters in flat, rigid chambers [17]. Here we employ our modeling approach to explain our new experimental findings.

## Methods

### Experimental procedures

Jurkat cells were grown and prepared for observation essentially as described before [11], [13]. Taxol and nocodazole (Sigma, St. Louis, MO) were dissolved respectively in DMSO and ethanol as described in manufacturer's manual and added to the cells suspended in RPMI 1640 growth medium (Invitrogen, Carlsbad, CA) at 1 µM and 100 nM respectively. Following the addition of the drugs the cell suspensions were preincubated for 30 min at 37°C and under 5% CO2. Control cells were treated identically except that pure solvent was added instead of the drug solutions. After the preincubation, the suspensions were transferred to poly-L-lysine-coated glass coverslips (BD Biosciences, Bedford, MA), which had been additionally coated with anti-TCR antibodies (clone UCHT1, Pharmingen, San Diego, CA) as described [11]. Each experiment was done in a controlled triplicate.

After 40 min of incubation on the coverslips (37°C, 5% CO2), the cells were fixed for 30 min at room temperature in 4% paraformaldehyde (Sigma), permeabilized in 0.5% Triton (Sigma) for 5 min, and blocked with 10% goat serum (Zymed, San Francisco, CA). Immunostaining was done with anti-α-tubulin mouse antibody (Molecular Probes, Eugene, OR) and goat anti-mouse TRITC-conjugated antibody (Zymed). The coverslips were mounted using ProLong mounting medium (Molecular Probes). The samples were observed on a Nikon TE 200 inverted microscope (Nikon, Melville, NY). A planapochromatic 100× oil-immersion objective with numerical aperture 1.4 (Nikon) was actuated by a PIFOC 721 piezo-positioner (Physik Instrumente, Auburn, MA). Images were acquired using a CARV II spinning-disk confocal attachment (BD Biosciences) and an ORCA II ERG camera (Hamamatsu Photonics, Bridgewater, NJ). All hardware was controlled by IPLab software (Scanalytics, Rockville, MD), which was also used for image manipulation. Three-dimensional images were acquired at a formal resolution (voxel size) of 0.129, 0.129, and 0.4 µm in the X, Y, and Z dimensions.

The cells were scored and classified for the centrosome polarity and centrality by examining the three-dimensional confocal images. The position of the centrosome was determined as the point of convergence of the fluorescent microtubules, usually corresponding to the point of maximum brightness. The cells were considered polarized if they displayed the microtubule aster converging within the bottom 2 µm of the cell, i.e. within 2 µm form the stimulatory substrate [12]. Otherwise they were considered as “non-polarized”. The polarized cells were further classified according to whether the centrosome was within 2 µm of the margin of the area of the cell contact with the substrate (“peripheral location”) or anywhere farther away from the margin (“central location”). All cells in 30 full-frame, random fields of view were scored for each sample. The results obtained in individual experiments within the triplicate were averaged, and the standard deviation (S.D.) between these results was calculated. The control groups from all experiments were pooled together after the absence of significant variation between them was ascertained.

### Computational methods

The method of computational prediction of the T-cell structure used in this work was essentially the same as described earlier [13], except for the newly-defined parameters of effective microtubule rigidity *κ_eff_* and simulated microtubule number *N*_sim_ (see below). Our approach is an extension of the microtubule aster optimization method proposed by Holy et al. [17]. Briefly, the T cell structure is modeled as consisting of an aster of microtubule fibers and of the bounding cell surface. Every microtubule has one of its ends in the same point in space, which point is termed the model centrosome; the other end is free. At the centrosome the ends are assumed to be clamped so that when unstrained, they would emanate from it in equally spaced directions in three dimensions. Then it is postulated that the cell structure seen in the experiment is the one corresponding to the minimum of an empirical energy function. This function penalizes microtubule bending, using the experimentally measured value of microtubule flexural rigidity. It also penalizes the cell surface area, using the experimentally measured value of leukocyte cortical tension (which has the dimension of surface tension). Further, this additive energy-cost function penalizes the deviation of the cell volume from a set value, using the known cell and tissue oncotic (macromolecular osmotic) pressure. Finally, the energy is considered reduced by the area of the contact between the T cell and the target surface, with a negative cost factor equal to an existing estimate of the two-dimensional energy density of receptor-mediated cell adhesion. A standard algorithm (sequential quadratic programming) from the Matlab Optimization Toolbox (Mathworks, Natick, MA) is employed to find the conformation of the model microtubule cytoskeleton which, when enclosed tightly in the cell surface, will result in the minimum value of the empirical energy function.

The optimization is performed in two stages, due to the limitations of the minimization algorithm. At the first stage, the optimal conformation of the microtubule cytoskeleton, without regard to its orientation as a whole, is found using an energy function that does not incorporate the term that describes the cell-target contact area. At the second stage, conversely, the proper conformation of the microtubule cytoskeleton is kept constant, and its orientation as a whole is found by minimizing the energy function that includes the attachment area term. The two stages of the algorithm may appear to model first a T cell that is freely suspended in the medium and then a T cell that develops contact with the stimulatory substrate. Despite this appearance, breaking down the computation into the two separate stages of finding the conformation and orientation is merely an empirical method of solving the optimization problem specified by our energy-minimization postulate about the T cell structure. This stepwise optimization method allows successful prediction of the structure and orientation of a conjugated T cell [13], which has not yet been possible in a dynamic model.

**Figure 1:**
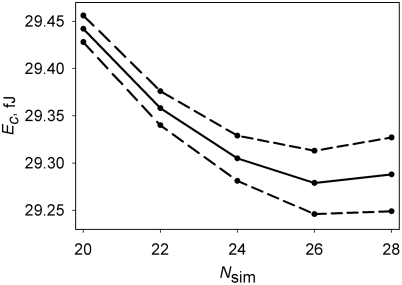
Dependence of the cell conformation energy that is achieved by the minimization algorithm on the number of segmented chains representing the microtubule cytoskeleton. The physical idealization of the cytoskeleton here has *N* = 88 microtubules, each having length *L* = 12 µm and flexural rigidity *κ* = 24 aJ µm. In the numerical algorithm, the rigidity *κ_eff_* of each of the *N*_sim_ chains is set to satisfy *N*_sim_ *κ_eff_* = *N κ* as *N*_sim_ is varied. Dashed lines, the 95% confidence interval. Based on 300 minimization runs for each point that are independent in the sense of the pseudorandom starting conformation.

At the first stage of the optimization procedure, each microtubule is approximated numerically as a chain of straight, freely jointed segments of equal length and number. The surface of the cell is defined geometrically as the minimum convex hull enclosing all the microtubules. The entire model cell structure is therefore determined by the set *X* of the direction angles of all microtubule segments (two angles, azimuth and declination, for each segment). An aster of straight microtubules is used as the starting conformation, randomized by adding a pseudorandom angle between 0 and 0.1 radians to each element in *X*. The sequential quadratic algorithm of the Matlab Optimization Toolbox (Mathworks, Natick, MA) is tasked to find *X* that minimizes the following conformation energy function, *E_c_*:
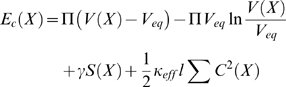



The first two terms describe work of cell volume change, done against the constant oncotic pressure of the tissue or medium (first term) and against the intracellular oncotic pressure (second term) that changes with the cell volume due to impenetrability of the cell boundary to macromolecules. Π = 3.4 fJ µm^−3^ is the oncotic pressure characteristic of mammalian tissues [18], and *V_eq_* = 2.1 pL is the goal cell volume consistent with the characteristic size of the cells in our experiments [13]. *V*(*X*) is the value of the variable cell volume corresponding in the above geometrical sense to the microtubule conformation specified by *X*. *S*(*X*) is the cell surface area. When multiplied by the leukocyte cortical tension *γ* = 35 aJ µm^−2^ [19], it yields the work of cell surface expansion, the third term in our cell-conformation energy function. *C*(*X*) is the local curvature of a microtubule, as determined by the microtubule segment directions in *X*. Squared and summed over all segment joints, then multiplied by the segment length *l* and by the effective microtubule bending rigidity *κ_eff_*, it yields the microtubule elastic bending energy. This is the last term of our empirical energy function. *l* equals the microtubule length *L* divided by the number of segments into which a microtubule is broken down. This number was selected to be 6 [13]. We represent the microtubule cytoskeleton consisting of *N* microtubules by a considerably smaller number of the segment chains in the simulation (*N*_sim_). Each chain has the effective flexural rigidity *κ_eff_* correspondingly higher than the rigidity of a single microtubule *κ*:
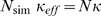
This allows setting *N*_sim_<*N* to reduce the number of independent variables (elements in *X*), which is crucial to the success of the numerical minimization. The approximation of the microtubule cytoskeleton with the smaller number of more rigid chains of segments can be valid if the chains are sufficiently numerous to represent adequately the shapes of all microtubules in the cell (it is assumed that microtubules are not bundled). A numerical test shows that *N*_sim_ = 24, which value we employed previously [13] without rigorous testing, is sufficient, because the results of the energy minimization cease to depend on *N*_sim_ beyond this number (Fig. 1). In view of this optimal value of *N*_sim_, for numerical convenience we select *κ* = 24 aJ µm from the range of experimental values reported for microtubule flexural rigidity (see [20]). The Matlab minimization routine is run until it converges, after which the cell structure is “shaken” by again adding the small pseudorandom angle to every element in *X* as was done in the beginning. The cycle is repeated until no further minimization can be achieved. The “shaking” is employed to make sure the minimization is not “stuck” in an insignificant local minimum. The described minimization of *E_c_* is computationally the most costly part of our algorithm. It requires about 1 h of processor time “per cell.”

At the second stage of the optimization procedure, the microtubule cytoskeleton conformation that was obtained at the first stage is kept fixed. The contact area with the target, *S_c_*, is determined by projecting the microtubule aster onto the horizontal plane that passes through the aster's lowermost point. The area of the minimal convex polygon enclosing the projection is considered to be the area of contact with the target. The cell surface is then recalculated as the minimum convex hull that encloses this projection (cell contact patch) as well as the microtubule aster (cell body). The cell structure depends now on only two variables, the two rigid-body rotation angles *θ* and *ϕ*. The optimal orientation of the aster is found as the *θ*, *ϕ* pair that minimizes the following orientation energy function, *E_o_*:
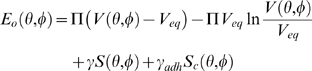
Compared to the goal function used at the first step in the optimization, the now-constant bending energy term is omitted here, and the new term of the negative (favorable) attachment surface energy is added. It is equal to the product of the contact area *S_c_* and the mid-range estimate of the two-dimensional energy density of cell adhesion (non-specific plus receptor-mediated), *γ_adh_* = −25 aJ µm^−2^ [21]. The microtubule cytoskeleton orientation corresponding to the global minimum of this function is found in Matlab by the previously described Monte Carlo method [13].

Our minimization algorithm overall remains local because of the local nature of the first stage and the very large number of variables at that stage. (There are 288 elements in *X* at the chosen level of numerical approximation of the microtubule cytoskeleton.) Starting from the pseudorandom initial structures, the two-stage algorithm returns predicted cell structures that are non-identical. We consider them all to be alternative predictions of the cell structure, postulating that the origin of individuality of the microtubule cytoskeletons seen in the experiment lies in the existence of multiple energy minima. The outcomes of individual runs of the minimization algorithm are therefore referred to as computational, or predicted, “cells” in this paper.

For characterizing the centrosome orientation in the computational cell with respect to the contact that this cell forms with the target surface, we are using the following angular measure. The cell centroid is found by approximating the predicted microtubule cytoskeleton by a three-dimensional ellipsoid in the least-squares sense. The angle formed with the vertical (perpendicular drawn to the underlying target plane) by the direction from the cell centroid to the centrosome is called the centrosome orientation angle *α*. This angular measure ranges from 0 for centrosomes pointing directly at the target to 180° for centrosomes pointing directly away from the target. The 0 and 180° directions correspond to “down” and “up” in our experimental model as well as in our computational convention.

## Results and Discussion

### Initial experimental findings

**Figure 2:**
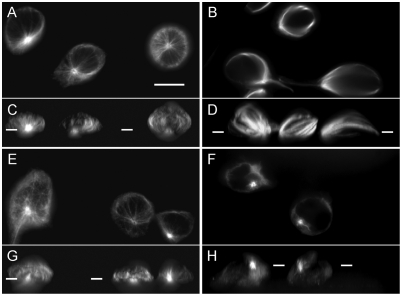
Microtubule cytoskeleton orientation in the experiment. Indirect immunostaining of tubulin in Jurkat cells attached to the stimulatory substrate. Representative fields of view are shown (see Table 1 for the statistics). For each experimental condition, a horizontal confocal section is shown in the panel above the panel containing the side view (a projection of the three-dimensional image). The level of the horizontal optical section is indicated by bars in the corresponding side-view image. The planar substrate under the cells is nonfluorescent. The centrosome is at the point of convergence of the fluorescently labeled microtubules, and therefore can alternatively be recognized as the brighter area in the image of each cell. The scale bar in *A* is 10 µm long. *A*, control cells, horizontal section. *B*, cells treated with 1-µM taxol, horizontal section. *C*, control cells, side view. *D*, cells treated with 1-µM taxol, side view. *E*, cells treated with 100-nM nocodazole, horizontal section. *F*, cells treated with 1-µM nocodazole, horizontal section. *G*, cells treated with 100-nM nocodazole, side view. *H*, cells treated with 1-µM nocodazole, side view.

Following Kuhne et al. [12], we considered a cell's centrosome as polarized if it was found within 2 µm from the stimulatory substrate, i.e. within a small fraction of the cell height. According to this criterion, essentially all Jurkat cells polarized their centrosomes within 40 min, whether they were untreated or treated with 1-µM taxol (Fig. 2A–D, Table 1). The conception that microtubule dynamics is important in T-cell centrosome polarization, which draws upon the perceived analogy with microtubule rearrangements in mitosis and on results of signal transduction studies [4], [5], is not compatible with this result. At the same time our new result is in agreement with the earlier experiments with 1-µM taxol, which were conducted on primary cytotoxic lymphocytes polarizing toward target cells [6]. Our result obtained in a different experimental model confirms that microtubule dynamics is not essential for T-cell centrosome polarization.

**Table 1:** Position of the centrosome in the experiment (the percentage is the fraction of the total number of cells).

Treatment	Polarized centrosomes, %, mean±S.D.	Peripheral centrosomes, %, mean±S.D.	Number of cells
Untreated	97.7±1.3	13.7±10.9	1842
1 µM taxol	98.4±1.2	79.1±2.8	669
100 nM nocodazole	89.2±0.7	11.3±3.1	545
1 µM nocodazole	25.6±6.1	19.5±8.6	322

At the same time we noticed a novel effect of 1-µM taxol on centrosome positioning. Only about 14% of untreated cells had their centrosomes at the periphery of the area of the cell's contact with the substrate. The same proximity criterion was used to classify the cell as having a peripheral location of the centrosome as for determining the polarization to the substrate: the centrosome's location was called peripheral if it was within 2 µm from the outline of the cell-substrate contact area. In contrast to the small fraction of untreated cells with the peripheral centrosome location, most cells treated with taxol exhibited the peripheral location of the centrosome 40 min after contacting the substrate (Fig. 2BD, Table 1). It is important to notice that the centrosomes that we call peripheral were still polarized to the substrate according to the proximity criterion, and that they were still positioned within the zone of the cell's “synapse” with the substrate.

To our knowledge, this effect of taxol on the centrosome positioning in T cells has not been reported before. Re-examination of the published structure of a T-killer cell conjugated with a target cell after taxol treatment [6] showed that it was visually consistent with our new finding. The taxol-induced shift of the typical orientation of the T-cell centrosome is potentially significant for the T-cell function. The simultaneous but differential secretion of immunological mediators in the direction of the target cell and “bystander” cells [22] could be modulated by the degree of proximity of the main secretory apparatus to the margin of the synaptic area. Firstly, the taxol-induced perturbation suggests that the exact position of the centrosome *within* the synaptic area may normally be under cellular control. Secondly, this perturbation may have direct implications for the therapeutic use of taxol, which will be discussed below.

To test whether the peripheral centrosome localization was a consequence of inhibiting microtubule dynamics, we determined the centrosome position in cells treated with another microtubule dynamics inhibitor, nocodazole at 100 nM. This treatment did not have any effect either on polarization of the centrosome to the substrate or on the proportion of cells with centrosomes at the periphery of the cell-substrate contact zone (Fig. 2EG, Table 1). From these results we conclude that the peripheral localization of the centrosome in the taxol-treated cells was likely caused by other effects of taxol than the inhibition of the microtubule dynamics as such.

Although mechanisms of action of microtubule drugs are complicated, it is generally accepted that there is a significant difference between the action of micromolar taxol and nanomolar nocodazole. Micromolar taxol stabilizes microtubules by shifting the assembly-disassembly balance greatly in favor of assembly [23], [24]. In contrast, nanomolar nocodazole inhibits assembly as well as disassembly, without inducing dissolution of the entire microtubule cytoskeleton that is characteristic of micromolar nodocazole concentrations [25], [26]. (Taxol also affects microtubule rigidity, although both increase and decrease of rigidity have been reported, depending on the experimental conditions, e.g., in [27], [28].) In view of the established difference between taxol and nocodazole action, we were led to hypothesize that the induction of the peripheral polarized position of the centrosome in our experiments specifically by taxol could be due to lengthening of microtubules caused by the assembly-promoting action specific to micromolar taxol and not to nanomolar nocodazole. There is no intuitive explanation, though, for how microtubule lengthening could cause the peripheral and at the same time polarized location of the centrosome.

### The model reproduces the two subpopulations of untreated cells

To determine whether the transition to the preferred peripheral location of the centrosome could be explained by microtubule lengthening by taxol, we resorted to our method of predicting the orientation of the T-cell microtubule cytoskeleton [13]. The method by design does not incorporate microtubule dynamics, but it has the static microtubule length as a parameter (see Materials and Methods). As detailed in Materials and Methods, we characterize the centrosome orientation by an angular measure, for which 0 is straight towards the substrate, i.e. toward the center of contact area, and 180° is straight away from the substrate. Thus angles above 90° mean that the centrosome is not polarized to the substrate at all, and angles around 45–60° indicate that the centrosome is pointed at the edge of the cell-substrate contact area.

**Figure 3:**
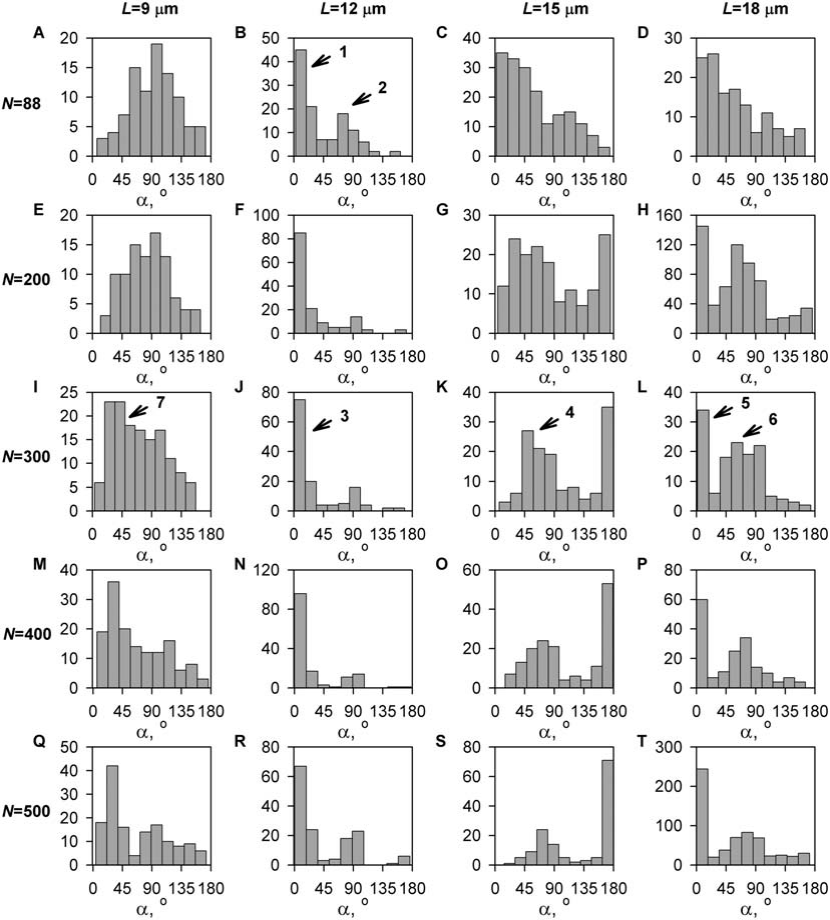
Distributions of predicted centrosome orientations for the indicated microtubule number (rows) and length (columns). α = 0 means the ideal functional orientation centrosome-down, and α = 180° means the opposite, non-functional orientation with the centrosome away from the stimulatory substrate. Numbers with arrows indicate histogram bins, sample microtubule cytoskeletons from which are shown in the correspondingly labeled parts of Fig. 4.

The new predictions of the centrosome orientation at different values of microtubule length and number in the cell are presented in Fig. 3. The histograms in this figure show that centrosome orientations between 0 and 180° are predicted in varying frequencies under different values of microtubule length and number. In general, the entire range of orientations is predicted to be populated for each parameter combination, but there are distinct peaks in the histograms, indicating that separate classes exist into which the most probable orientations fall. Most commonly, more than one peak is predicted for the given length-number condition. This feature corresponds well to our impression from visual inspection of microscopic images: that there are distinct classes of cells and that the peripheral location of the centrosome, for example, is not merely a limit of a continuum of centrosome locations but a distinct class of cell structures (cf. Fig. 2). Admittedly, quantitative demonstration of this impression as well as of the other basic microscopic observations regarding the cell structure will be prohibitive, considering that many hundreds of three-dimensional images will need to be measured in detail (as with the numbers of the real and numerical cells in Table 1 and Fig. 3).

**Figure 4:**
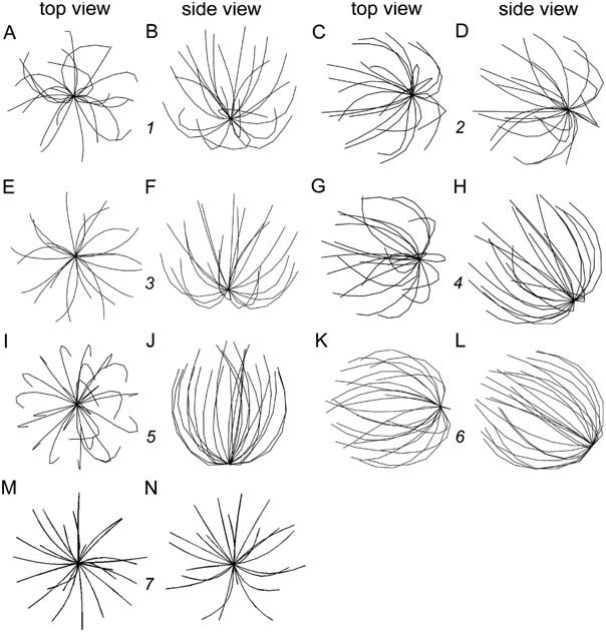
Sample predicted microtubule cytoskeleton structures and orientations. Only 24 microtubules are drawn in each computer-generated image, according to the three-dimensional shapes specified by the numerical algorithm. Top and side views of each sample structure are shown next to each other. Orientation centrosome-down is the ideal functional orientation in our plotting convention, to match our experimental setup. The number between the top and side view of the same structure corresponds to the number of the peak as labeled in Fig. 3. *A* and *B*, the major type of structure predicted when there are 88 microtubules 12 µm long. *C* and *D*, the minor type of structure predicted when there are 88 microtubules 12 µm long. *E* and *F*, the dominant type of structure, when there are 300 microtubules 12 µm long. *G* and *H*, the dominant type of structure, when there are 300 microtubules 15 µm long. *I* and *J*, the minor type of structure predicted when there are 300 microtubules 18 µm long. *K* and *L*, the major type of structure predicted when there are 300 microtubules 18 µm long. *M* and *N*, the major type of structure predicted when there are 300 microtubules 9 µm long.

We have previously found that the numerical optimization condition equivalent, in the terms adopted in the present work, to having *N* = 88 microtubules in the cell that are each *L* = 12 µm long predicts adequately the orientation of the normal (untreated) T cell [13]. The new prediction of the distribution of orientations in the entire population of cells that corresponds to this number-length condition is presented in Fig. 3B. The histogram shows that the orientations near the zero angle indeed predominate. An example of a predicted cell structure belonging to this dominant mode in the distribution is plotted in Fig. 4AB. It reasonably approximates the typical structure and orientation of the microtubule cytoskeleton in the predominant type of untreated cells (cf. two of the three cells in the field shown in Fig. 2AC). Specifically, the centrosome appears pointing down in the side view and is in the middle when viewed from the top.

The new and more extensive computations reveal a significant second peak in the orientations distribution around 75° (Fig. 3B). The existence of the second, minor peak is remarkable in the light of the new experimental data indicating that a minor fraction of untreated cells exhibit a distinct peripheral location of the centrosome at the interface with the substrate. A predicted cell structure belonging to this second mode of the distribution is shown in Fig. 4CD. It approximates the secondary type of structures seen among untreated cells in the experiment (cf. one of the two cells in the field shown in Fig. 2AC). Specifically, the centrosome appears pointing down as well as to the side in the side view and is eccentric when viewed from the top.

The prediction departs from the experiment in two ways. First, the relative weight of the two peaks predicted under this number and length of the microtubules deviates noticeably from the experiment. By the number of predicted cells that fall into these peaks, the two subpopulations comprise approximately 62% (the 0° mode) and 38% (the 75° mode). Thus, there are more of the non-central centrosomes in this predicted population than in the actual untreated cell population (cf. Table 1). Furthermore, their modal 75-degree orientation begins to approach 90°, beyond which angle the centrosome cannot be actually considered polarized to the substrate. The predictions under the previously chosen conditions of the microtubules length and number therefore appeared not entirely satisfactory, when not only the dominant mode but the entire sample of predicted cell structures were considered on the quantitative level. Yet these predictions matched the experiment reasonably well insofar as they reproduced the number of cell structure classes, their kind, and their relative prevalence in the untreated cell populations. This degree of approximation of the untreated cell population was deemed sufficient as a starting point for constructing explanation of the qualitative transition observed in the taxol experiment, which we then attempted.

### Lengthening model microtubules reproduces the major subpopulation of taxol-treated cells

Computation shows that increasing the length of the microtubules while keeping their number the same as previously considered (*N* = 88) only blurs the distinction between the two orientation modes without shifting their relative weight (Fig. 3B–D). Thus starting from the previously considered combination of microtubule parameters for the untreated cell, the model failed to explain the taxol-induced change in centrosome orientation distribution by lengthening of the microtubules within the reasonable range.

The failure led us to consider a higher number of microtubules in the T cell. It is generally believed that microtubule numbers are in the hundreds in typically studied cultured cells (see [29]), and we are aware of no direct indication that they should be less numerous in Jurkat cells. It is important to point out that the linear structures seen in images such as Fig. 2 are not likely to be individual microtubules. Rather, these visible lines should represent variations of spatial density of the much more numerous microtubules under the limit of optical resolution. Such variations would be correlated along the locally common direction of the microtubules. This effect would create the impression of linear structures in the image that are far less numerous but have the same general orientation as the actual microtubules. An in-depth discussion of this effect in the case of the actin cytoskeleton can be found in [30]. Indeed, the directions of microtubules were discerned by early microscopists as “rays” running from the centrosome, when the visibility of individual microtubules was out of question considering the microscopy techniques used (e.g., [31]). Also, considering the total cellular concentration [32], [33] and polymer-monomer partition [34], [35] of tubulin, the microtubule lattice length per subunit [36], and the cell volume and microtubule length assumed here, it can be calculated that there may be as many as 500 microtubules in our model cell.

The second column of panels in Fig. 3 shows that increasing the number of microtubules while keeping their length the same as previously assumed (12 µm) makes the comparison of the predicted orientation distribution with the untreated experimental cell population more favorable in that the weight of the secondary peak is reduced. This is observed with intermediate numbers of microtubules, 200 to 400. Notably, the secondary peak is becoming more populated again with a further increase in microtubule number to 500 (Fig. 3R). Thus, there is a realistic range of microtubule numbers in which the real population of untreated cells is matched by the computational model, with regard to the centrosome orientation in the major subpopulation of cells. At the same time, the modal orientation of the minor subpopulation increases with the microtubule number (Fig. 3, second column). This makes comparison with the experiment less favorable in that the centrosomes from the minor subpopulation of cells are becoming less polarized to the target surface, pointing instead more to the side of the cell (close to 90°). Despite the inaccuracy of the minor distribution mode, the cell structures from the major mode still resemble the major subpopulation of untreated cells very closely. A computational cell structure belonging to the dominant mode of the cell population predicted at 300 12 µm-long microtubules is plotted in Fig. 4EF (cf. the experimental image in Fig. 2AC). In fact, it is superior to the prediction made with 88 microtubules (Fig. 4AB) in that not only does the centrosome “point” to the cell bottom, but it now lies even lower in the microtubule aster (Fig. 4EF).

Considering the 200–400 microtubules 12 µm long now as the base conditions, we increased the microtubule length in the simulation, attempting to reproduce the hypothesized effect of taxol. Fig. 3 shows that an increase in microtubule length from 12 to 15 µm was sufficient to make most populated the distribution peak that was secondary before. This shift is very clear for microtubule numbers of 200 to 300 (Fig. 3). The most numerous subpopulation under these conditions (200–300 microtubules 15 µm long) has a modal centrosome orientation of about 45° (Fig. 3GK). This orientation signifies that the centrosome is aimed at the edge of the contact area with the target surface. As an example, a computational cell structure from this mode of the orientational distribution is plotted in Fig. 4GH. It resembles the dominant cell structure type seen in the taxol-treated experimental population (cf. Fig. 2BD), specifically in that the centrosome is oriented to the bottom as well as to the side. Thus the computational model supports the hypothesis that lengthening of microtubules under the action of taxol, not the suppression of the microtubule dynamics per se, can be responsible for the distinctive preferred orientation of the microtubule cytoskeleton that is observed in the experiment.

### Realistic microtubule lengthening reproduces the minor taxol-treated subpopulation as well

The prediction at the 15-µm microtubule length in the 200–300 range of the microtubule number is problematic in that the orientational distribution under these conditions does not exhibit a secondary peak at the “normal” 0° orientation (Fig. 3). Instead, the secondary peak is predicted at the completely non-polarized, 180° orientation (Fig. 3). This part of the prediction contradicts the experiment (Fig. 2, Table 1). It must be noted that we have previously considered a mechanism that may be able to actively eliminate cells in the “incorrect” orientation from the population of cells attached to the substrate [14], so that they would not be observed in the fixed-cell type of experiment from which the data in Table 1 have been obtained. In addition, there are other mechanisms such as active intracellular translocation [37] and whole-cell movement driven by dynamic receptor density gradient [15] which, at least in principle, may convert the non-polarized cells to polarized ones to some degree. Nonetheless, the prediction of the present model departs in this parameter regime from the experiment qualitatively.

The 1.25-fold increase in the microtubule length (from 12 to 15 µm) should not be the largest possible increase that could be induced by the taxol treatment. The taxol concentration used is essentially saturating [6], [24], and there is normally about as much tubulin in the microtubules as there is in the monomer pool [34], [35], which could be shifted into the polymer (microtubule) pool by promoting assembly. We therefore felt justified in increasing the microtubule length in the model further, to see if this brings the prediction in closer agreement with our experiment.

Increasing the microtubule length to 18 µm (a 1.5-fold increase from the base value of 12 µm) preserves the dominant 45–90° peak in the centrosome orientation distribution in the range of the microtubule lengths where it was predicted with the 15-µm microtubules, especially in the 200–300 microtubule number range (Fig. 3). At the same time, the peak at the non-polarized 180° orientation is not prominent with the 18-µm microtubules (Fig. 3). Instead, the “normal” 0° orientation again becomes a prominent mode of the distribution with the 18-µm microtubules, just as it was with the 12-µm microtubules. While prominent, this peak is very narrow, and it is therefore a minor fraction of the total predicted cell population (Fig. 3). This prediction, with 200–300 18 µm-long microtubules, provides the best match for the results of our taxol experiments.

There is a trade-off in the accuracy of the predictions between 200 and 300 microtubules. The tertiary mode of non-polarized (180°) cells is entirely absent with 300 microtubules, as it appears to be in the experiment. Its absence however is achieved at the cost of the cells with their centrosomes oriented to the side (90°) being more numerous than with 200 microtubules (Fig. 3). Structures resembling neither 180° nor 90° theoretical orientation are seen with any appreciable frequency in the experiment. These tertiary subpopulations seen in the theoretical distributions are, however, rather minor (Fig. 3), and the prediction with either 200 or 300 microtubules can be considered satisfactory overall.

Finally, we illustrate the theoretical explanation of the taxol experiments using the model predictions at 300 18 µm-long microtubules (Fig. 4I–L). The peak to which most predicted cells belong has under these conditions the modal centrosome orientation of approximately 60° (Fig. 3). This orientation means that the centrosome in the attached cell is oriented towards the substrate and to the side. An example of the cell structure from the mode of this peak (Fig. 4KL) closely resembles in this respect the dominant type of cell structure seen in the taxol-treated experimental cell populations (cf. Fig. 2BD). A sample cell structure from the 0° mode in the same predicted distribution is shown for comparison in Fig. 4IJ. It reproduces well the less prominent structural class seen in our taxol experiments (Table 1).

In the light of the present model (not implicating the above possibilities that other previously modeled effects might be involved), the mismatch between the model and experiment assuming 15-µm microtubules and the good match between them assuming 18-µm microtubules can be taken to indicate that the microtubule length in taxol-treated T cells exceeds 15 µm and is more likely to be near 18 µm. To arrive at this estimate, the above comparison of the model and experiment can be viewed as data-fitting. The qualitative changes in the shape of the centrosome orientation distribution as the microtubule length is varied continuously (Fig. 3) are an intriguing feature of the model. However this type of abrupt and nonlinear dependency of the predictions on the parameter values is common in adequately complex models of biological phenomena other than the so far less studied predictive models of cell structure. It would be interesting to attempt verification of the model prediction obtained with intermediate microtubule length (15 µm) by developing a method for more precisely controlling the degree of tubulin polymerization with intermediate doses of microtubule-stabilizing drugs. Similarly it would be interesting to attempt verification of the above data-fitting estimation of the microtubule length in high concentration of taxol (18 µm) by developing a method for reliably resolving and tracing individual microtubules in experimental images to determine their length directly.

### Test of the model against experiments with shortening the microtubules

To further validate the ability of the model to predict consequences of microtubule length change, we have calculated the distributions of the centrosome orientation assuming that the microtubules were only 9 µm long (the leftmost column of histograms in Fig. 3). The model predicts substantial randomization of the distribution: absence of the “normal” peak at 0° and broadening of any peaks that remain. The effect is particularly significant in the range of microtubule numbers between 200 and 300, which range reproduced the effect of taxol in the previous calculations, as well as below this range (*N* = 88). To test the prediction of the orientational randomization by microtubule shortening, we conducted experiments with nocodazole in micromolar concentrations. In agreement with the previous studies [25], [26], [38], these high concentrations of nocodazole (1 µM) caused visible shortening of the microtubules and dramatically inhibited polarization of the centrosome to the stimulatory substrate in our experimental system (Fig. 2FH, and Table 1). A discrepancy between the model and experiment can be noticed as to the degree of eccentricity of the centrosome in the cell (cf. Fig. 2FH and Fig. 4MN). However there is a good match between the theory and experiment in that the preferential orientation of the centrosome to the substrate is lost when the microtubules are shortened (cf. Fig. 3, leftmost column, and Table 1). These results confirm the capability of our model to infer centrosome orientation from microtubule length, which capability was employed above to explain the novel effect of taxol.

### Conclusions and outlook

The results of our experiments with taxol and nanomolar nocodazole confirm the conclusion from earlier experiments with taxol on primary cytotoxic T-lymphocytes that microtubule dynamics is not required for the immunologically functional orientation of the centrosome in T cells [6]. These new and previously published results of experiments in which microtubule dynamics was directly modulated with inhibitors are at odds with the speculations that microtubule dynamics as such may be involved in T cell polarization, which speculations find support only in T-cell signaling studies [4], [5]. The results of the direct microtubule dynamics inhibition also argue against drawing analogy between T-cell polarization and microtubule rearrangements in mitosis. In its insensitivity to abrogation of microtubule dynamics by taxol, polarization of T-cell centrosomes to the target, rather, parallels other types of microtubule rearrangements characteristic of leukocytes: during initiation of migration in neutrophils [39] and during retraction into the uropod in motile T cells [40].

Our experiments reveal a subtler effect of micromolar taxol but not of nanomolar nocodazole on the centrosome positioning in polarized T cells. Micromolar taxol promoted peripheral localization of T-cell centrosomes within the contact zone of T cells with the target surface. It should be emphasized that these centrosomes are still at the interface with the target and are in this sense polarized functionally. At the same time this effect appears potentially very significant in the emerging framework that recognizes T cells as sending spatially differentiated signals to the target and “bystander” cells [22]. It is conceivable and merits experimental investigation that the peripheral-synaptic centrosome localization results in spatially mixed signaling, in which case its representation in the cell population will be important for the overall degree of signal segregation. The observed contrast between the control and taxol-treated cell populations indicates that the centrosome position may normally be under tighter control than merely ensuring its proximity to the target. At the same time, absence of the centrosome displacement from the synapse center in cells treated with nanomolar nocodazole indicates that the more precise positioning of the centrosome within the synapse does not require microtubule dynamics either.

The results of our computational modeling demonstrate that the effect of taxol on centrosome orientation can be rigorously explained by lengthening of the stabilized microtubules, which is specific to the micromolar taxol [23]–[26]. Our computational modeling approach assumes static microtubule length. Thus, even the secondary effect of taxol on centrosome positioning in T cell can be explained without invoking suppression of the microtubule dynamics as such. This rigorous theoretical result lends further support to the notion that microtubule dynamics per se (i.e. the stochastic assembly and disassembly of individual microtubules) are not involved in the functional centrosome polarization in activated T cells, not even in the finer aspects of the centrosome positioning.

Another effect of taxol on microtubules is promotion of acetylation [41]. The present biomechanical model deals only with cell-scale structural properties of the microtubules, such as their number and length. It cannot be used to study the potential effect of a biochemical modification such as the acetylation. Therefore, even though our model rigorously explains the peripheral centrosome localization as being a consequence of microtubule elongation, it does not rigorously refute the theoretical possibility that the peripheral centrosome localization may rather be a consequence of the microtubule acetylation.

Successful explanation of the subtler effects of experimental treatment on the overall microtubule cytoskeleton structure argues in favor of employing the cell structure optimization method more broadly in cell biology. It carries obvious advantages when it is the cell structure that needs explaining, and when dynamic simulations predicting the structure would necessitate more specific assumptions about quantities and mechanisms not firmly established experimentally.

It should be pointed out (discussed in detail in [13]) that not making specific assumptions as to the origin of the affinity of the T cell to the target, the energy-minimization method does not negate specificity of the antigen-mediated immunological cell interactions. The method is at the same time indeed more broadly applicable, as it explains the centrosome polarization to the substrate that is seen in experiments in which T cells as well as other immune and non-immune cell types exhibit affinity to suitably prepared, immunologically nonspecific substrates [42]–[44].

In the light of the new experiments, however, success of the energy-minimization prediction method poses a new question. The original calculations of the conformational energy landscape by Holy et al. were made for microtubule asters confined in flat, rigid chambers [17]. They correctly predicted the eccentric positioning of the centrosomes in the experiment. These calculations, like ours, assumed static microtubule length as a parameter. The calculated energy landscape had local minima besides the global minimum that gave the correct prediction–also like in our model (see [13]). That the global minimum was reached in reality was ascribed by Holy et al. to the capacity of microtubule dynamics to facilitate conformational transitions [17]. What would make the minima accessible when the microtubule dynamics is experimentally suppressed? Our supposition [13] that it may be the stochastic variations in the actomyosin cortex (which is responsible for the cell surface tension, a factor in our model) merits further investigation now that the microtubule-dynamics alternative has been refuted. It must be mentioned, however, that there are experimental and theoretical models for other phenomena than T cell polarization in which microtubule dynamics does affect centrosome positioning (e.g., [45]–[47]). Also, molecular motors pulling on the microtubules can provide the source of stochasticity as well as play a more direct role in centrosome positioning, as suggested for T cells [37] and analyzed theoretically in the context of other cell-biological phenomena (e.g., [48], [49]).

Side effects of taxol as an anti-cancer drug on the immune system are widely known. They have been linked to its suppressing cell divisions that must replenish immune cells in the organism (e.g, [50]). Our findings raise the possibility that at least part of the side effects of taxol may be related to its modulating the orientation of the centrosome and the associated secretory apparatus in T cells during their immunological interactions with other cells.

Another line of speculation prompted by our findings is related to the fact that certain viruses attacking the T cells use the polarization of the centrosome-associated secretory apparatus during cell-cell interactions for direct propagation between cells. These include (reviewed in [51]) human T cell leukemia virus type 1 (HTLV-1) and human immunodeficiency virus type 1 (HIV-1). Subtle modulation of centrosome positioning in T cells by well-studied drugs such as taxol may potentially be used to reduce the efficiency of the virus spread in the body. It would be interesting to explore whether deliberate induction of the imperfect centrosome orientation by taxol may strike the right balance, impeding the virus spread sufficiently for it to be cleared by the immune response, but not disrupting the immunological function of T cells to a dangerous extent. Considerable success of the computational modeling approach used in the present work points to the possibility of precise computational predictions of cell-level effects of such therapeutic interventions, which could facilitate their rational development.
